# Complex study of air pollution in electroplating workshop

**DOI:** 10.1038/s41598-020-67771-3

**Published:** 2020-07-09

**Authors:** K. Yu. Kirichenko, I. A. Vakhniuk, V. V. Ivanov, I. A. Tarasenko, D. Yu. Kosyanov, S. A. Medvedev, V. P. Soparev, V. A. Drozd, A. S. Kholodov, K. S. Golokhvast

**Affiliations:** 1 Far Eastern Federal University, 8 Sukhanova Street, Vladivostok, 690950 Russian Federation; 2 Far Eastern Geological Institute, FEB RAS, pr-t 100-let Vladivostok, 159, Vladivostok, 690022 Russian Federation; 3 Joint Stock Company “Izumrud”, Vladivostok, Russian Federation; 4 Public Joint Stock Company “Dalpribor”, Vladivostok, Russian Federation; 5 Far Eastern Scientific Center of Physiology and Pathology of Breath SB RAMS, 73G Russkaya Street, Vladivostok, 690105 Russian Federation

**Keywords:** Environmental monitoring, Natural hazards

## Abstract

A comprehensive analysis of the state of air inside an industrial workshop with electroplating production was carried out. The data of quantitative distribution of suspended particles by size fractions (PM_0.3_, PM_0.5_, PM_1_, PM_3_, PM_5_, PM_10_) are presented for 15 main processes of electroplating. Morphometric and chemical composition of the surface of particles were studied. We observed particles of rounded shape, various agglomerates with complex geometric shapes, acute-angular particles, which when inhaled pose a maximum threat to human health. Chemical analysis of these particles showed an absolute predominance of oxides of non-ferrous metals, the percentage of which varied depending on the type of electroplating bath. The content of highly hazardous substances of the 1st (Zn, Pb, and Cd) and the 2nd (Cu, Cr, Ni, Co, and Mo) hazard classes in each sample was recorded.

## Introduction

Various industries (including defense and space enterprises) perform protective coatings by employing the process of electroplating. Electroplating involves working with chemically active solutions such as NiSO_4_; MgSO_4_; Na_3_PO_4_ and al., and heavy metals and is classified as a hazardous industry. According to the Russian Federal State Statistics Service (ROSSTAT), there is an annual increase of workers employed in jobs involving harmful and/or hazardous working conditions^1,2^. The constant impact of negative factors of electroplating production leads to occupational illnesses, mainly diseases of the respiratory system and upper respiratory tract, circulatory system, musculoskeletal system. These occupational illnesses have been observed in workers having work experience of 10 to 15 years. These statistics are associated with the formation of atmospheric particulates saturated with nano- and microparticles of heavy metals, as an essential attribute accompanying electrochemical processes.

Traditionally, much attention is directed to the treatment of wastewater saturated with heavy metal ions in workshops equipped with electroplating baths^3,4^, as pollution of the aquatic environment can be a factor hampering the country's socio-economic development^5^. In this regard, various recommendations were developed^6^ to rationalize emissions and reduce the anthropogenic load on the environment^7–9^. The study of air pollution in the working area, including nano- and micro-sized particulate matter (PM) in workshops and the adjacent territory, has been given less importance, despite the fact that these particles are detected at considerable distances from industrial buildings^10^. These particles produce a significant effect on climate formation processes in industrial centers^11^, where the proportion of anthropogenic particles reaches 45% of the total number of aerosol particles^12^. Timely assessment of significance and danger of nano-technological contamination can reduce socio-economic indicators and the level of public health^13^. In order to reduce the number of diseases and deaths, it is required to implement effective preventive and protective measures for workers of hazardous industries and employees of related professions. The solution to this problem is possible only after studying the characteristics of industrial aerosol particulates generated in an electroplating workshop. This paper deals with the analysis of the state of air environment inside an electroplating plant and a comprehensive study of morphometric parameters of nano- and microparticles formed at enterprises that use electroplating baths and apply basic electrochemical processes.

## Methods

### Measurement of the quantitative composition of PM

AeroTrak Handheld Particle Counter 9,306 (TSI Incorporated, USA) was used as a sampler. This sampler meets all the requirements set out in ISO 21501-4.

**Figure 1:**
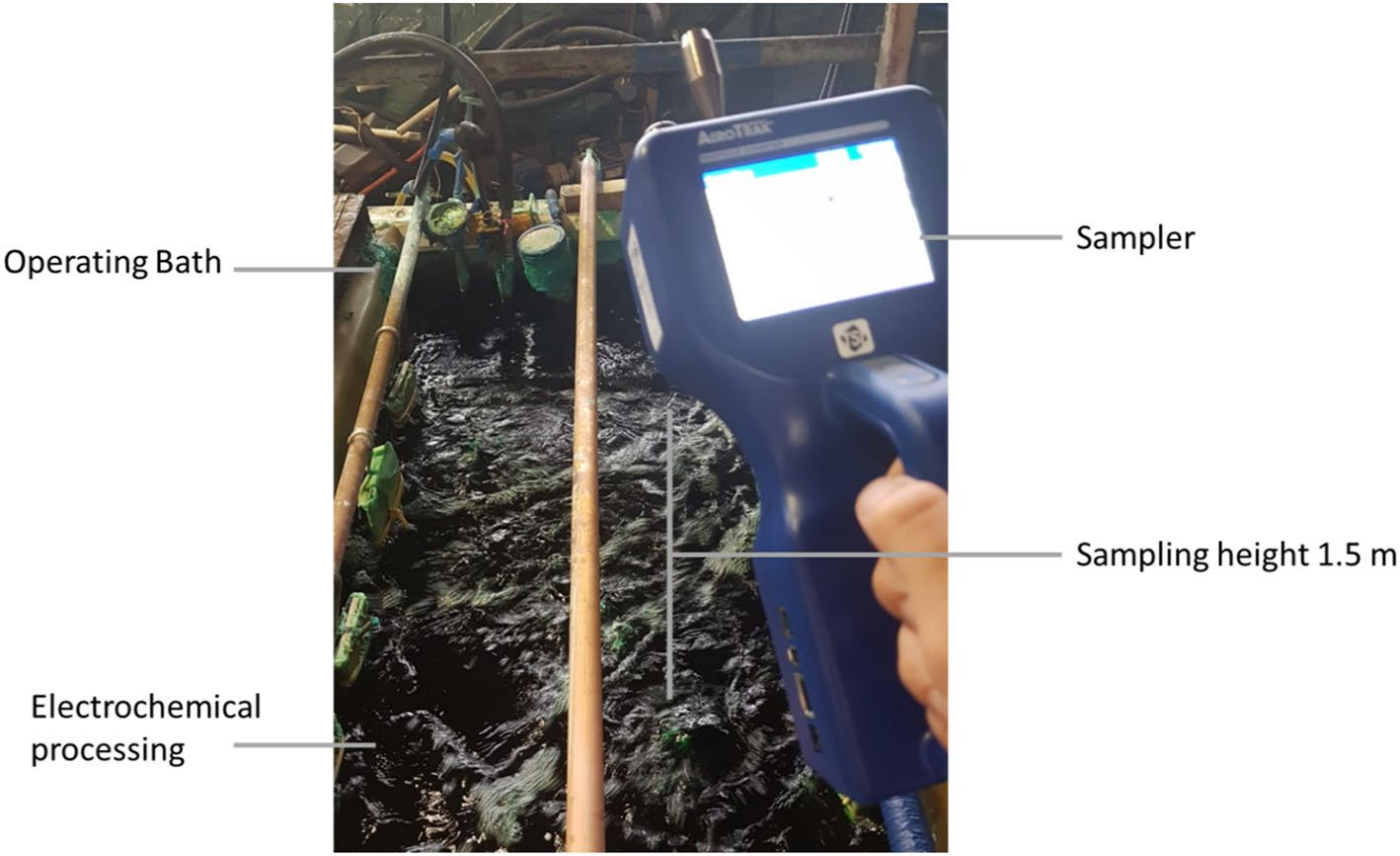
Sampling of industrial aerosol particles in the electroplating workshop. H = 1.5 m (Example).

The sampling time at each point was 1 min. The volume of air blown was 2.83 l per minute. The sampling height was 1.5 m, corresponding to the height of the average human breathing level (Fig. 1). Samples were taken near operating baths of the aluminum preparation line, non-ferrous metals preparation line, and protective coatings line. In total, 15 measurements for electroplating baths with various uses were made during a series of experiments. For the first time, we comprehensively studied aerosol particles from the most common electrochemical processes in the industry—electroplating (Table 1).

**Table 1:** Sampling for measurement of particle size distribution.

No	Sampling point	Electrolyte composition
1	Aluminum cleaning	HNO_3_
2	Aluminum etching	NaOH
3	Sulfuric acid anodizing	H_2_SO_4_
4	Aluminum degreasing	Na_2_CO_3_; Na_3_PO_4_
5	Chemical degreasing	Labomid 203
6	Cathodic degreasing 3	Na_2_CO_3_; Na_3_PO_4_
7	Cathodic degreasing 4	Na_2_CO_3_; Na_3_PO_4_
8	Cold rinse	H_2_O
9	Desmutting	Cr_2_O_3_; H_2_SO_4_; NaCl
10	Nonferrous metals etching	HNO3; H_2_SO_4_; HCl
11	Chromium plating	H_2_CrO_4_; H_2_SO_4_
12	Nickel plating	NiSO_4_; MgSO_4_; Na_2_SO_4_; NaCl; H_3_BO_3_
13	Chemical nickel plating	NiSO_4_, 7H_2_O, NaH_2_PO_2_, CH_3_COOH
14	Cadmium plating	CdO; NaCN
15	Silvering	AgCl, K_2_CO_3_, AgNO_3_, K_4_[Fe(CN)_6_]

### Measurement of particle size distribution

The sampling procedure was as follows: 2.7 l sterile plastic containers with distilled water were placed on the floor of the workshop during its operation. Containers were filled to 1/3 of the total volume (600–800 ml) with distilled water obtained on the DE-4-02-EMO water distillator (Electromedoborudovanie, St. Petersburg, Russia). The still bath name (process), date and time were recorded for each sample.

The experiment duration was eight hours which is equal to a shift in the workshop. The containers were placed near still baths and opened at 8.00 a.m. with the beginning of the work shift. At the end of the work shift at 5:00 p.m., the containers were tightly closed, marked and transported to the laboratory for further research. A total of 15 samples were taken (Table 1).

The samples were transported to the Research and Educational Center Nanotechnology at the School of Engineering FEFU. In the lab, the samples were dispersing in an ultrasonic bath to homogenize the settled particles and then 100 ml of liquid were collected from each sample for analysis on Analysette 22 NanoTec plus laser particle analyzer (Fritsch GmbH, Berlin, Germany). Each sample was analysed thrice in two modes i.e. nano (0.01–45 μm) and micro (0.08–2,000 μm). The measurement results were calculated by the Mie-Grüneisen equation of state.

### Measurement of mass concentration of the particles

A series of samplings was carried out near still baths in order to determine the content of fine particles of industrial aerosol in the working zone air of the electroplating workshop. The concentration of particles was measured with the gravimetric method using an aspirator-type sampler LVS 3.1 (Ingeniero Norbert Derenda, Germany). This sampler is equipped with an attachment for sampling PM_10_ particles and 47 mm nylon-based filters (Nylon 66 Membranes) with the working surface diameter of 47 mm (SUPELCO, USA). The filter capacity is 0.45 μm. Duration of sample collection was during the workday (8 h).The measured range of particulates is from 0.45 to 10 μm. The upper limit of detection—10 μm (PM_10_) was chosen because it represents the greatest hazard to human health, causing respiratory diseases^14–16^. Emphasizing PM_10_ fraction reflects the current trend in the monitoring of substances suspended in ambient air^17^.

Before the sampling procedure, the filters were pre-dried in a TC-1/20 thermostat (Russia) for 24 h at 40 °C, then each filter was weighed five times on Sartorius electronic scales (Germany), with arithmetic mean value determined.

In total, measurements were taken at seven points in the electroplating workshop (Table 2), the statistical sampling was three measurements for each point. An emphasis was placed on still nickel plating baths because these baths are the sources of radiation of particles which are considered as the most toxic for humans. The height of the sampler attachment corresponded to the level of human breathing—1.5 m.

**Table 2:** Particle concentration measurement points.

No	Process	Sampling distance (m)
1	Chemical nickel plating	1
2	Nickel plating	1
3	Nickel plating	3
4	Nickel plating	5
5	Aluminum etching	1
6	Cadmium plating	1
7	Silvering	1

### Electron microscopy of particulates

The research was carried out in the laboratory of micro- and nano-research of the Far East Geological Institute FEB RAS on two analytical scanning electron microscopes:

1. JSM-6490 LV (Jeol, Japan), equipped with an INCA energy dispersive X-ray spectrometer, X-max 80 and INCA Wave dispersive spectrometer (Oxford Instruments, Great Britain).
2. Lyra 3 XMH (Tescan, Czech Republic)—a dual-beam microscope (a combination of electron and ion columns in the single chamber) with a Schottky cathode (with field emission), equipped with an energy dispersive X-ray spectrometer AZtec, X-Max 80 Standart.

The specimens were sputtered with carbon beforehand. Approximate quantitative elemental analysis was carried out in reflected electrons (BSE detector), with a working distance of 9–10 mm and an accelerating voltage of 20 kV. The results were processed using qualitative and quantitative elemental analysis software—INCA Point & ID and AZtec.

## Results and discussion

### Measurement of quantitative composition of particulates

The results obtained indicate the absolute predominance of minute particles (PM_0.3_) (Table 3). The smallest particles of heavy metals from industrial aerosols are able to penetrate deeply into the human respiratory organs and further spread throughout the body^18^, causing the development of chronic diseases and a general decrease in efficiency. The number of particles less than 0.3 μm in diameter is over 10,000 times larger than the number of PM_10_ particles. The maximum number of 0.3 μm and 0.5 μm particles was recorded near the nickel plating bath (Table 1). This was a still bath used for electrochemical working of metals, equipped with ventilation systems and heating from a DC source, with the working temperature of 15–25 °C. The chemical composition of the bath was:

- According to Russian technical standard (nature abbreviation "GOST") No 2665–86 concentration of nickel sulfate should be in the range 140–200 g/l;
- GOST No 4523-77, concentration of magnesium sulfate is 30–50 g/l;
- GOST No 6318-77, concentration of sodium sulfate is 50–70 g/l;
- GOST No 9656-75, concentration of boric acid is 25–30 g/l;
- GOST No 4233-77, concentration of sodium chloride is 3–5 g/l;
- We should note a high level of particles with dimensions from 1 to 10 μm near the electroplating bath used for chemical degreasing of non-ferrous metal parts.

**Table 3:** Quantitative composition of industrial aerosol particles in the electroplating workshop.

No	Sampling point	Q-ty of PM_0.3_	Q-ty of PM_0.5_	Q-ty of PM_1_	Q-ty of PM_3_	Q-ty of PM_5_	Q-ty of PM_10_
0	Background-office	4,310,371	791,546	62,586	5,296	2,004	268
1	Aluminum cleaning	519,674,500	84,421,240	7,266,013	1,135,918	490,619	61,614
2	Aluminum etching	506,923,000	77,166,580	6,328,771	920,321	375,156	46,185
3	Sulfuric acid anodizing	506,473,800	84,629,070	7,742,597	1,300,552	566,366	79,331
4	Aluminum degreasing	774,991,600	162,161,400	9,128,154	969,094	398,746	47,678
5	Chemical degreasing	448,472,500	134,870,800	**30,231,220**	**12,399,540**	**7,378,788**	**1,195,740**
6	Cathodic degreasing 3	643,172,200	130,576,300	7,848,006	985,816	415,468	59,026
7	Cathodic degreasing 4	708,764,000	143,945,600	8,276,812	992,087	410,790	54,745
8	Cold rinse	538,953,100	92,534,710	6,765,838	901,209	370,179	44,991
9	Desmutting	554,218,000	82,453,290	6,413,975	836,908	335,341	43,199
10	Nonferrous metals etching	295,190,000	45,127,460	4,913,552	662,420	268,551	31,056
11	Chromium plating	546,641,900	99,608,620	9,115,911	916,737	332,952	31,852
12	Nickel plating	**844,536,600**^a^	**277,202,500**	19,315,880	614,841	178,968	10,949
13	Chemical nickel plating	170,112,500	19,012,500	3,270,000	673,929	309,286	41,786
14	Cadmium plating	328,474,600	45,382,330	3,261,131	365,724	157,951	25,442
15	Silvering	536,869,600	87,845,940	5,920,848	298,233	117,315	17,668

^a^Bold shows highest results.

Since the samples were taken directly above the contents of still electroplating baths, the results indicate fine dimensions of primary particles of industrial aerosols formed during the electrochemical processes. Most primary particles were less than 0.3 μm in diameter. The number of particles of this fraction was predominant for all studied electrochemical processes.

### Measurement of particle size distribution

**Figure 2:**
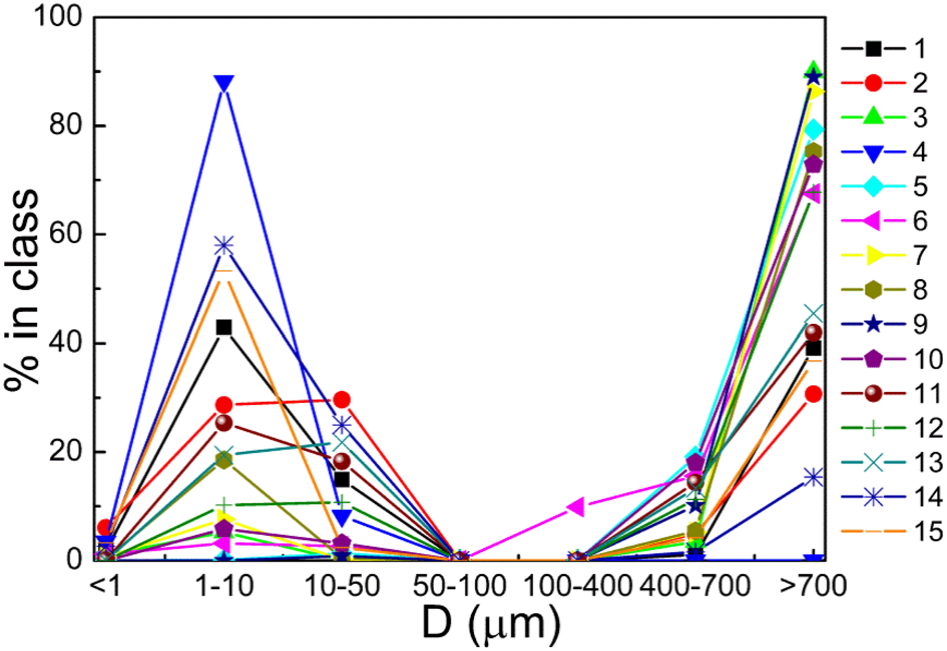
Comparison of size distribution of particles of electroplating origin from all processes.

The overall percent distribution of particles of electroplating origin using laser granulometry is shown in Fig. 2.

**Figure 3:**
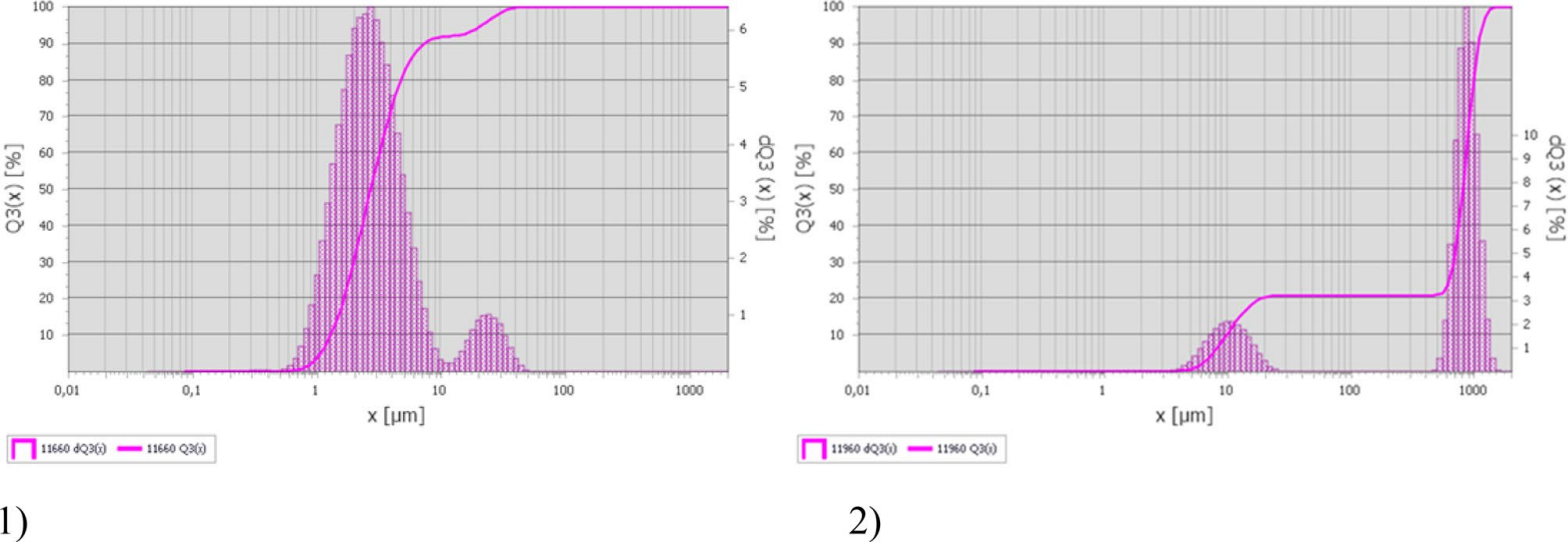
Size distribution of particles in sample 1) No. 4 (aluminum degreasing), and 2) No. 12 (nickel plating).

The presented figures show typical graphs of particle distribution based on the results of particle size analysis. Conventionally, the results obtained can be divided into two types of graphs; with the predominance of particles less than 10 μm in diameter (Fig. 3a), and with the predominance of particles greater than 700 μm in diameter (Fig. 3b).

In particularly the sample taken on the aluminum hardening line that was also mentioned above (Fig. 3a) contained the highest quantity of particles smaller than 10 μm in diameter in the electroplating shop—88.2%. This sample was taken near a still aluminum degreasing bath. Out of 15 collected samples, only four contained the predominant quantity of particles of this fraction, two of these samples were taken on aluminum hardening line, and the other two—on plating lines. At the remaining sampling points, the diameter of the predominant particles was higher than 700 μm, ranging from 30 to 90% (Fig. 3b). It is important to note that the particles in the range from 50 to 400 μm were almost completely absent in the samples (Fig. 2).

Quantitative analysis data (Table 1) showed the absolute predominance of particles with a diameter smaller than 10 μm, and the highest number of particles had dimensions under 0.3 μm, i.e. correspond to PM_0.3_ class. Size distribution data analysis (Fig. 2) demonstrated the quantitative predominance of larger particles as compared with the results of quantitative analysis obtained using the handheld particle counter. This discrepancy was due to the agglomeration of the smallest primary particulates in the working zone air of the electroplating workshop during the shift. During the work shift primary particulates with a diameter under 0.3 μm, forming over still baths, stick together to form larger aggregates and clusters, which, when they reach over 700 μm in diameter, are more prone to the law of gravity and begin to precipitate.

### Measurement of mass concentration of particulates

The results of measurement of the concentration of particulates with diameter under 10 μm are presented below (for 1 m distance from the source) (Table 4).

**Table 4:** Comparison of PM_10_ particles concentrations with air quality standards.

Title of process	PM_10_ (mg/m^3^)
Chemical nickel plating	0.04 ± 0.0001
Nickel plating	0.04 ± 0.0002
Aluminum etching	0.04 ± 0.0002
Cadmium plating	0.08 ± 0.0003
Silvering	0.04 ± 0.0002
Russian hygienic standard GN 2.1.6.2604-10	0.06
USEPA NAAQS^19^	0.05

According to the Russian Occupational safety standards on harmful substances (GOST 12.1.007-76 SSBT Harmful substances. Classification and general safety requirements), all chemicals are divided into four hazard classes:

- 1st hazard class: maximal permissible concentration (MPC) of harmful substances in the working zone air is below 0.1 mg/m^3^;
- 2nd hazard class: MPC is from 0.1 to 1 mg/m^3^;
- 3rd hazard class: MPC is from 1 to 10 mg/m^3^;
- 4th hazard class: MPC is greater than 10 mg/m^3^.

The obtained data on the mass concentration of particulates inside the electroplating workshop did not reveal concentrations exceeding the daily values of the MPC. The exception was the sample taken near the electroplating bath with the cadmium plating process. This fact can be related to the physical characteristics of the sampler, namely to the throughput rate of selected filters (0.45 µm), meaning that airborne particles with smaller dimensions freely passed through the aspirator and did not settle on the filter. Therefore, it may be assumed that when conducting an experiment with filters that have a lower throughput rate, the results obtained may exceed the MPC values. Although traditionally it is considered that, despite significant quantitative content, ultrafine particles make little contribution to the total mass of particulates^20^.

### Electron microscopy of particulates

The morphological structure of the largest nano- and microparticles is important for determining the level of their toxicity. For example, it was previously reported that dendritic and fusiform-shaped industrial particulates are more cytotoxic^21,22^. This is partly due to the fact that sharp thin edges of these particles can cause significant mechanical damage to cell membranes, which in turn leads to the death of cells.

Given below are the microimages of the structure of particles, and their energy dispersive analysis. Summary (Figs. 4, 5, 6, 7, 8, 9, 10, 11, 12, 13, 14, 15, 16, 17, 18) shows our data on the chemical composition of particles obtained at all 15 electrochemical processes. Note that such detailed chemico-morphological analysis for each type of electrochemical process in electroplating was performed for the first time.

**Figure 4:**
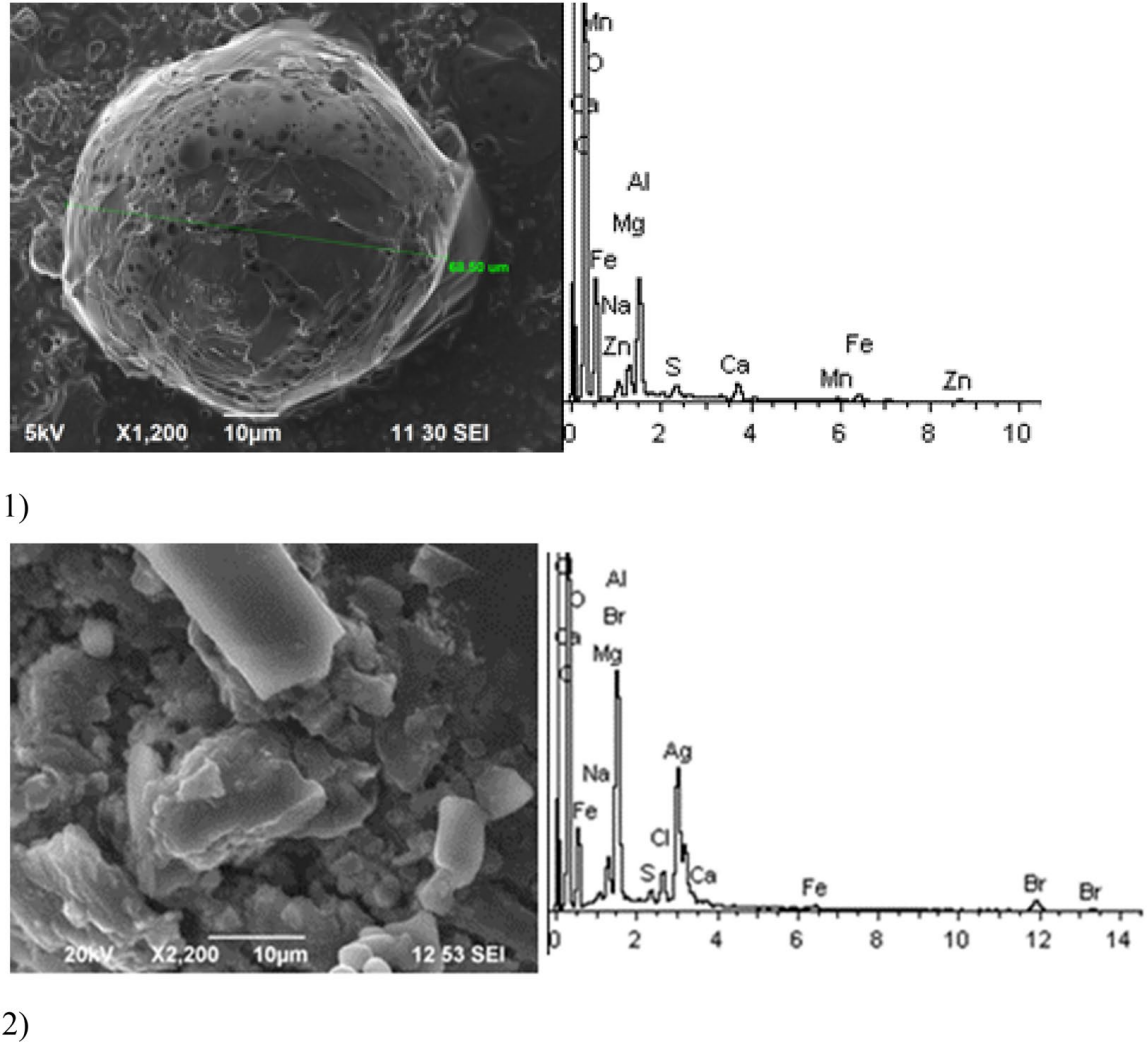
Aluminum cleaning. Particles morphology. Scanning electronic microscopy. 1) Magnification ×1,200, 2) Magnification ×2,200.

**Figure 5:**
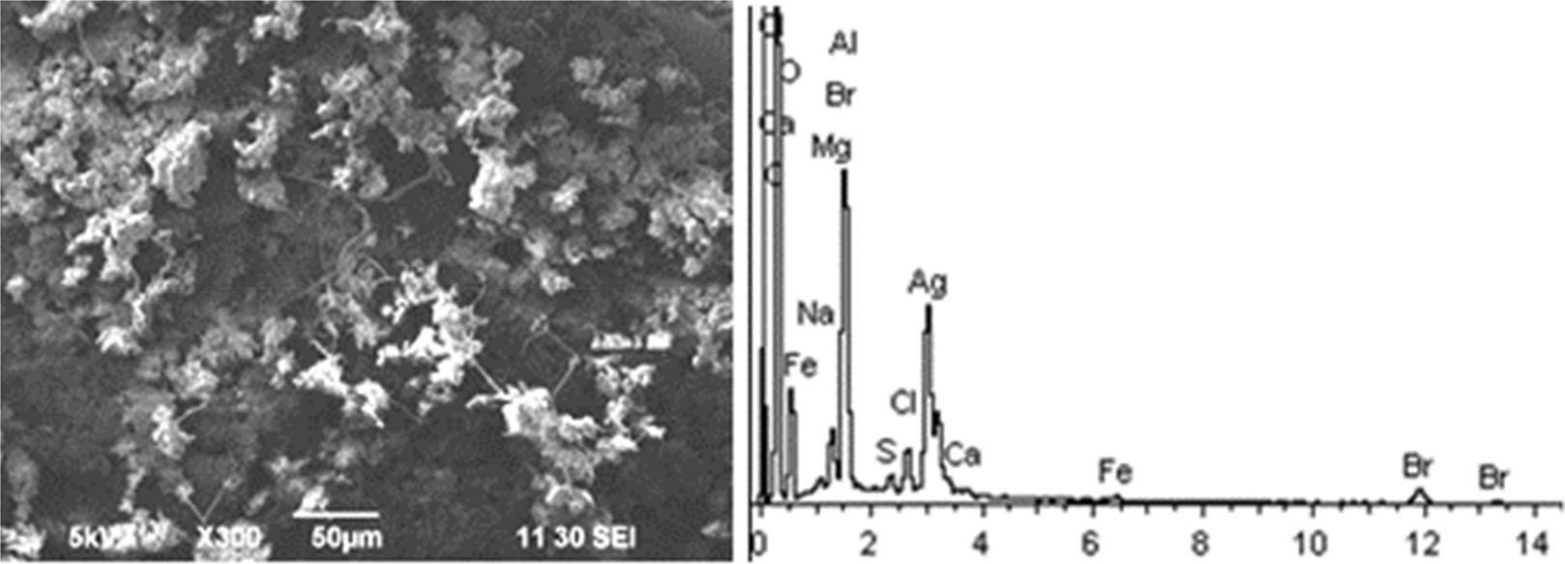
Aluminum etching. Particles morphology. Scanning electronic microscopy. Magnification ×300.

**Figure 6:**
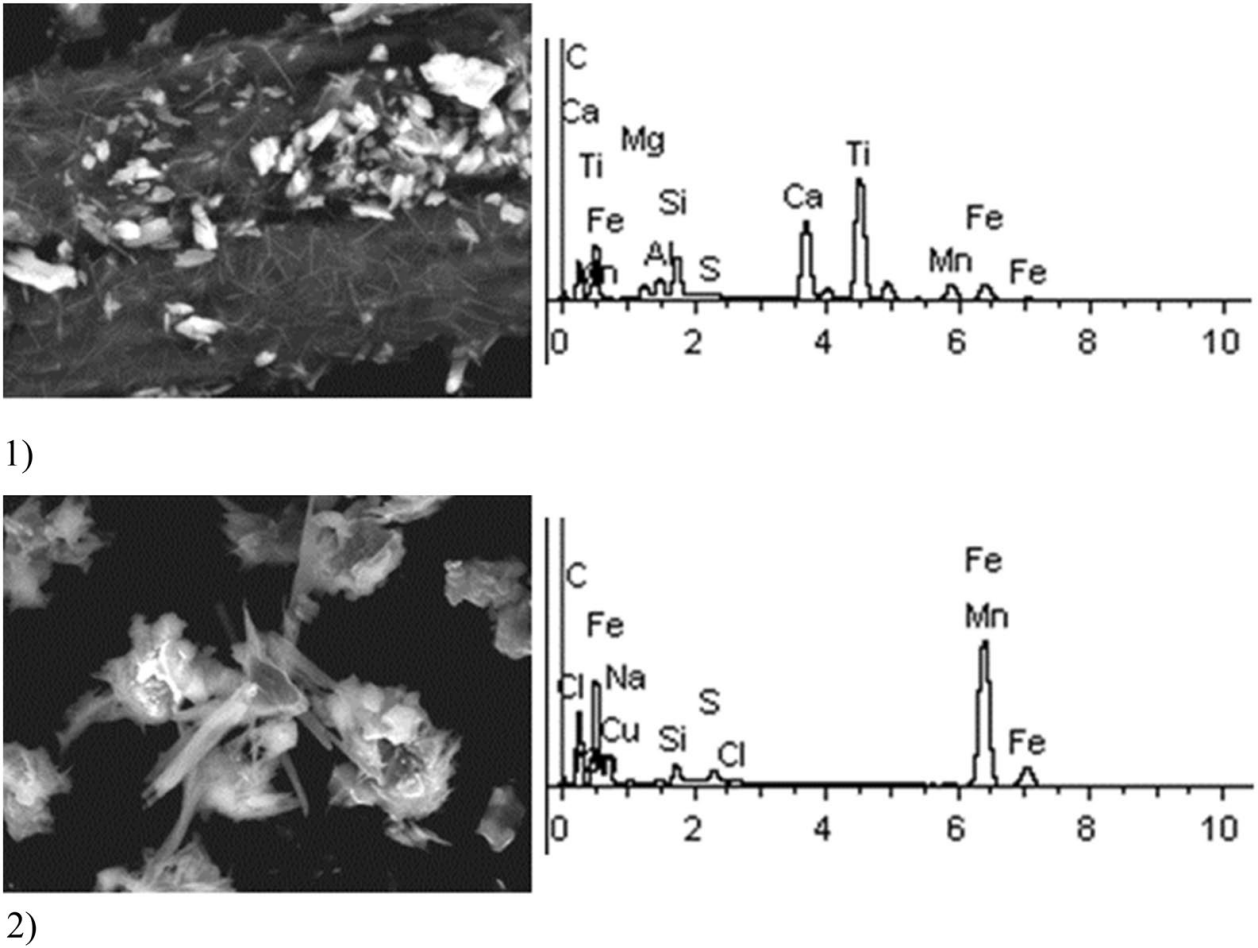
Sulfuric acid anodizing. Particles morphology. Scanning electronic microscopy.

**Figure 7:**
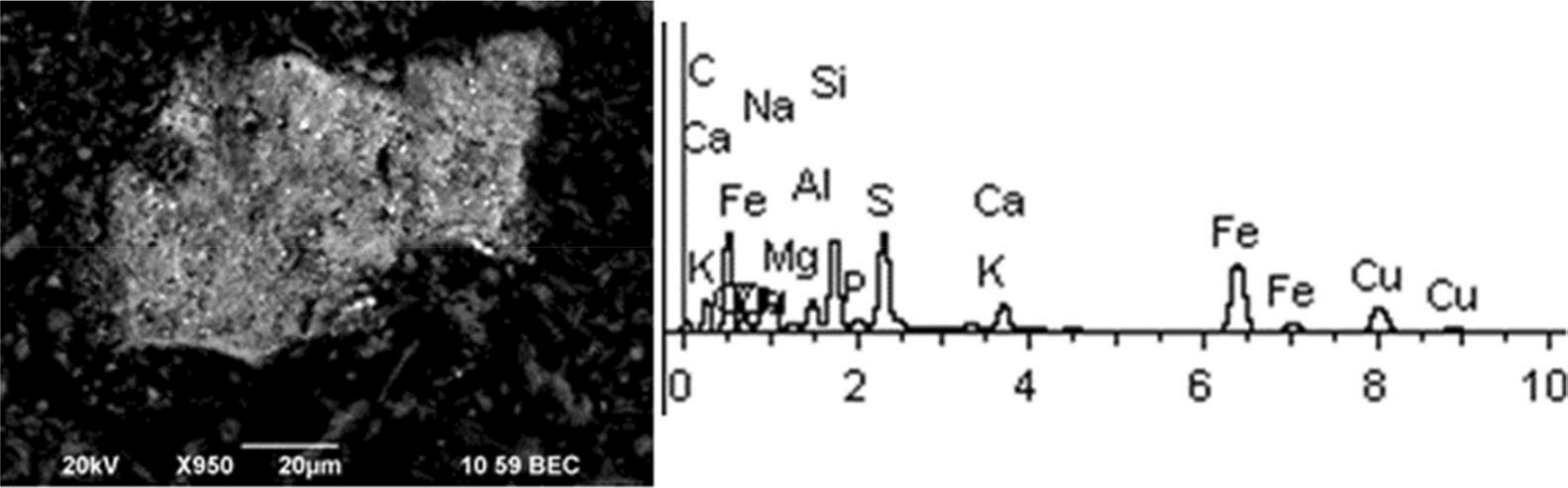
Aluminum degreasing. Particles morphology. Scanning electronic microscopy. Magnification ×950.

**Figure 8:**
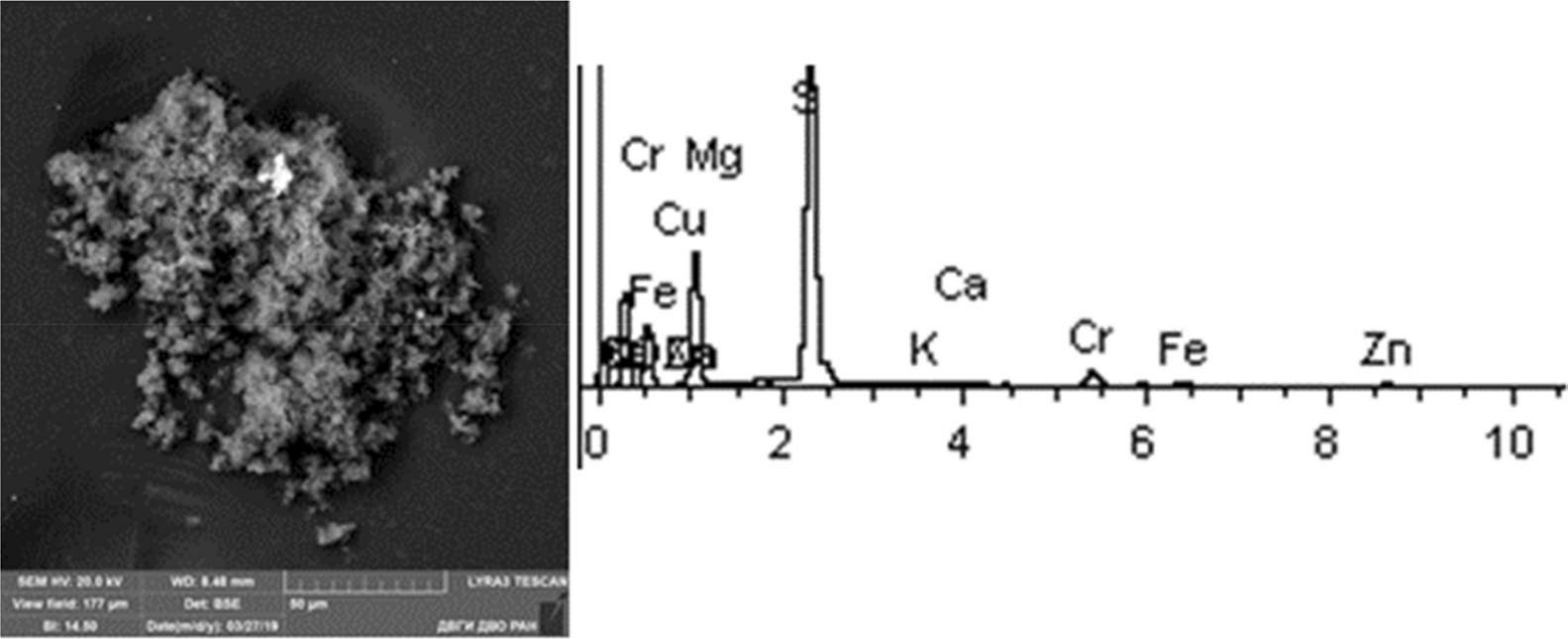
Chemical degreasing. Particles morphology. Scanning electronic microscopy. Measuring interval 50 µm.

**Figure 9:**
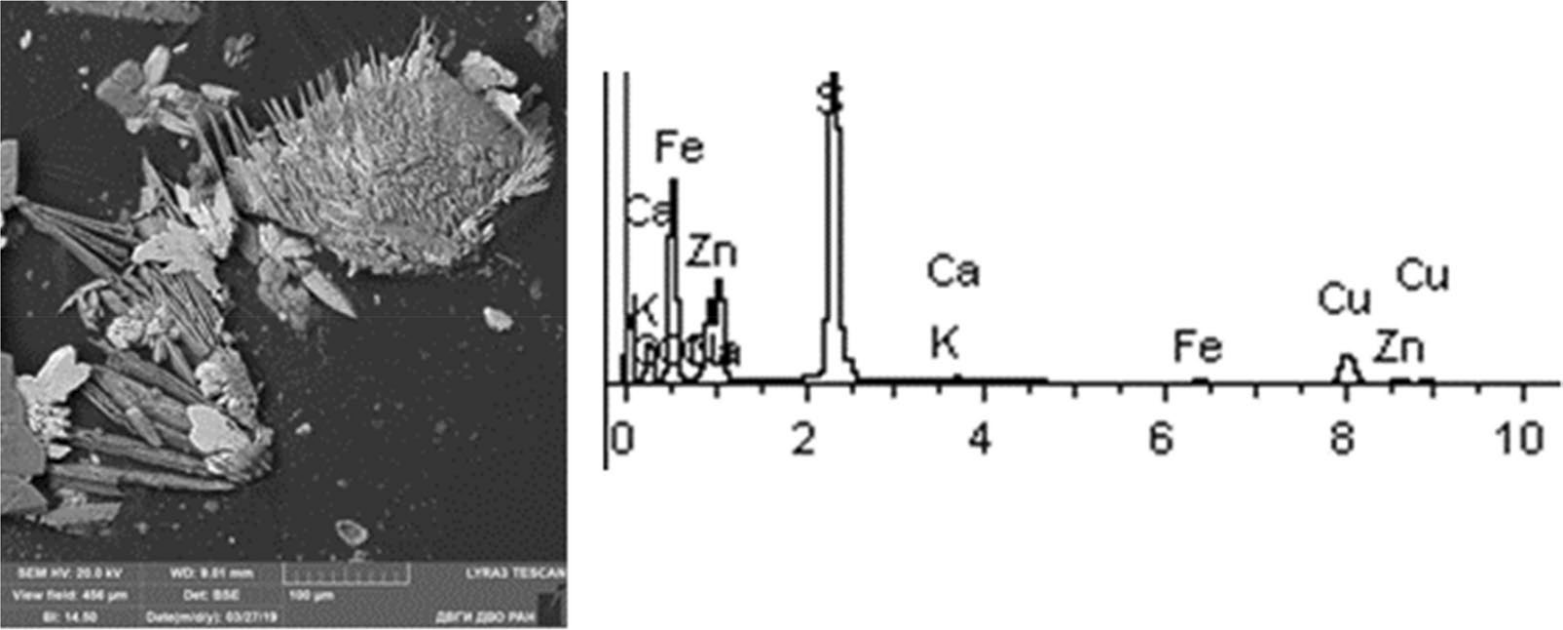
Cathodic degreasing 3. Particles morphology. Scanning electronic microscopy. Measuring interval 100 µm.

**Figure 10:**
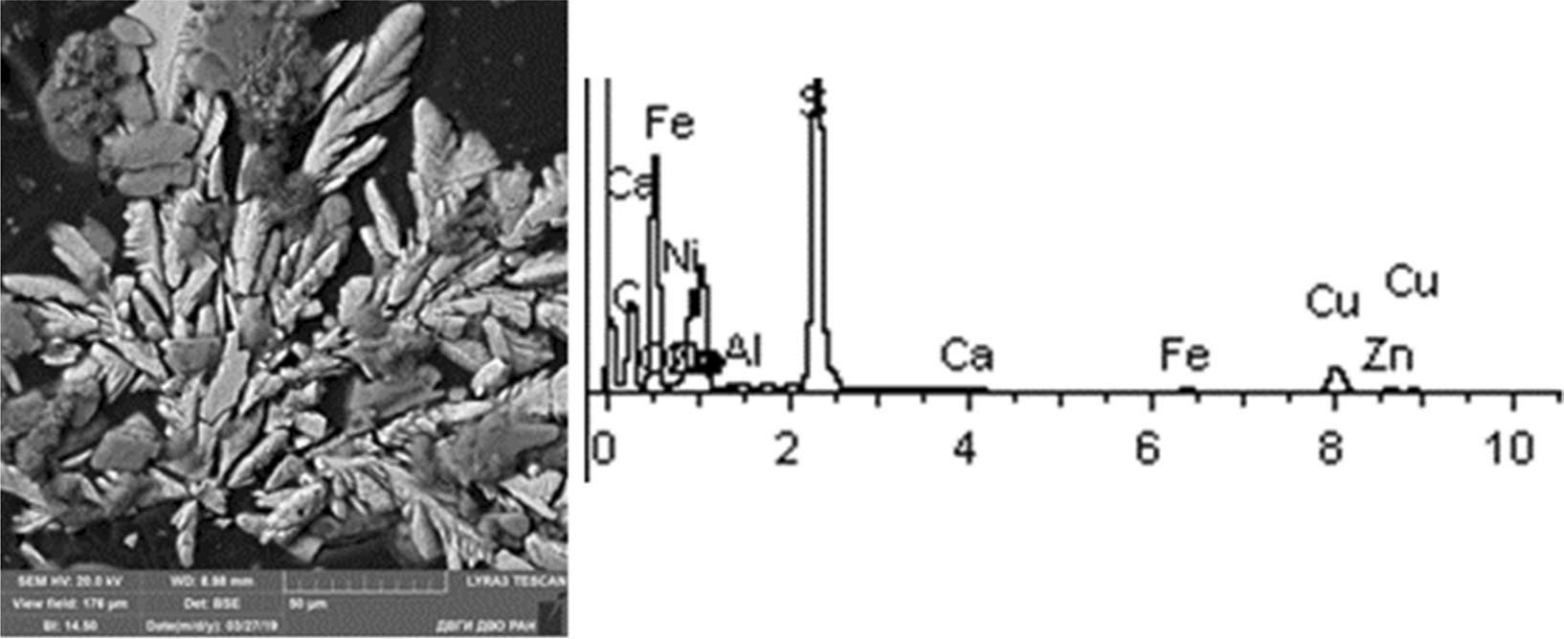
Cathodic degreasing 4. Particles morphology. Scanning electronic microscopy. Measuring interval 50 µm.

**Figure 11:**
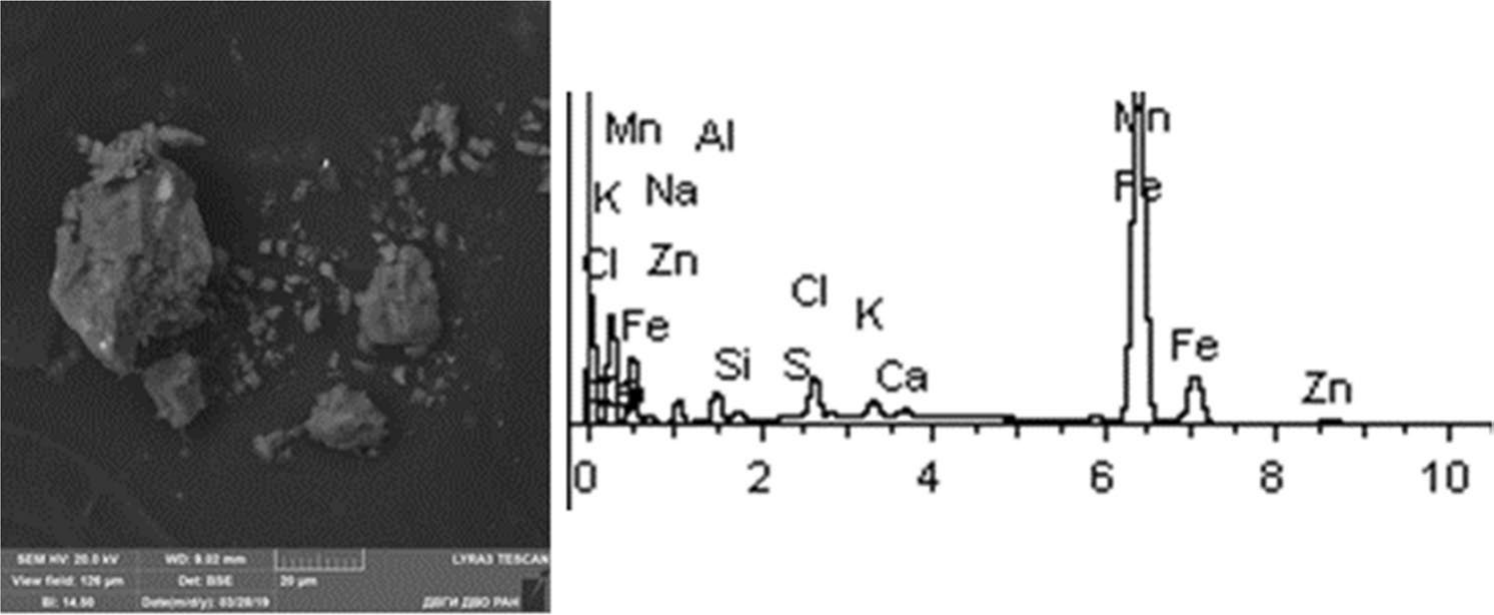
Cold rinse. Particles morphology. Scanning electronic microscopy. Measuring interval 20 µm.

**Figure 12:**
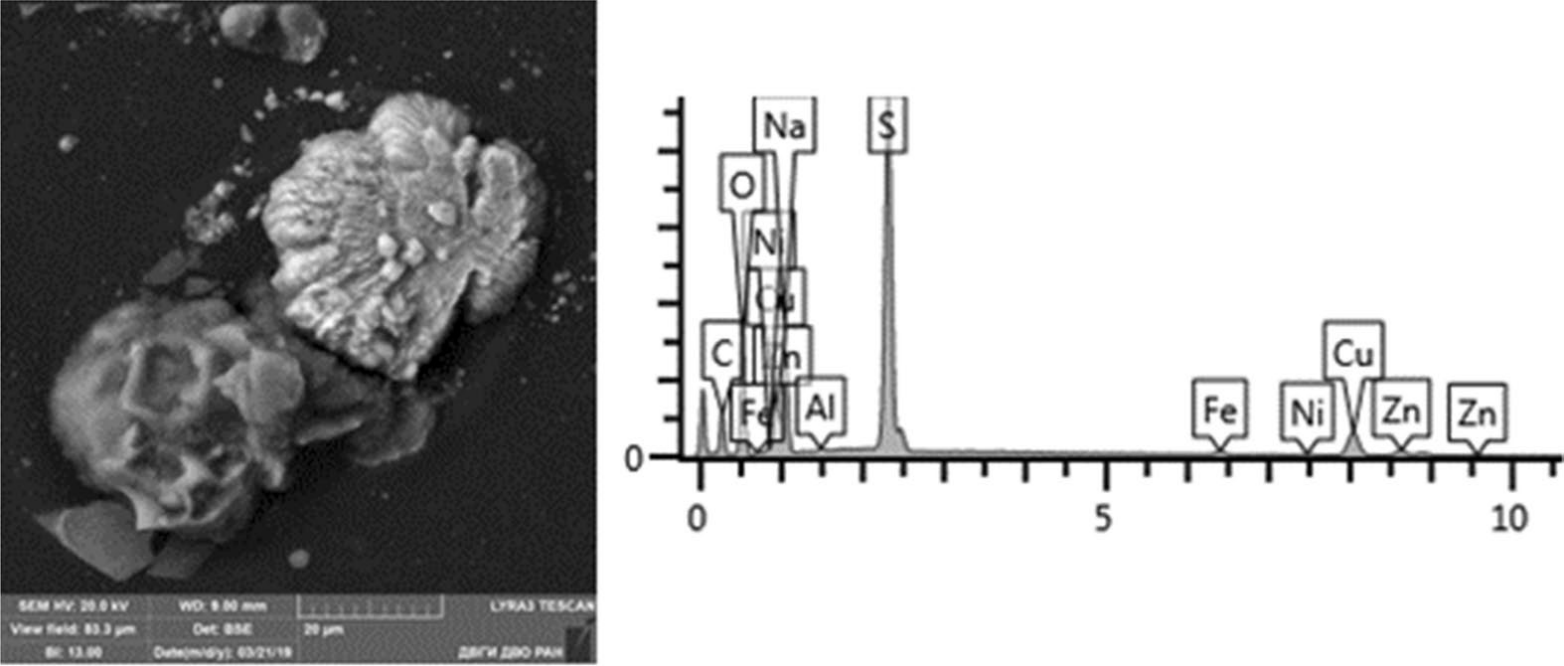
Desmutting. Particles morphology. Scanning electronic microscopy. Measuring interval 20 µm.

**Figure 13:**
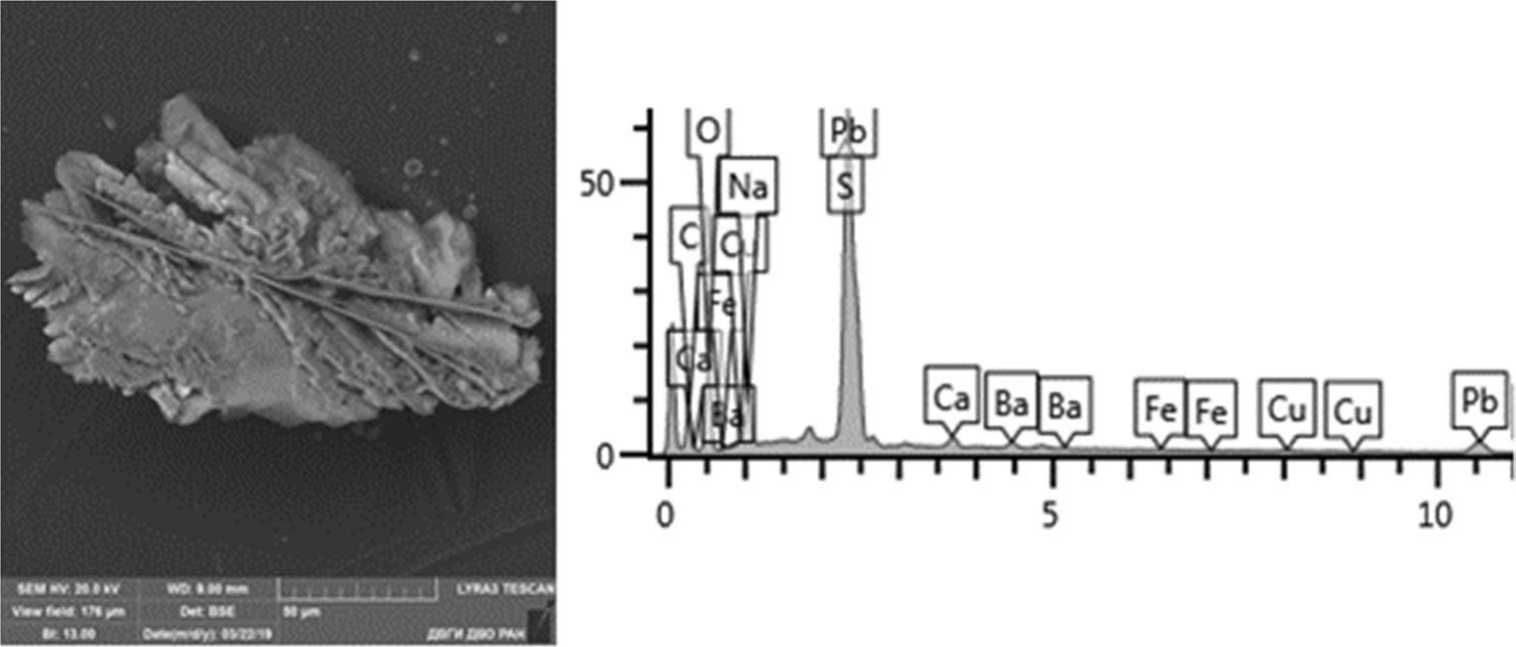
Nonferrous metals etching. Particles morphology. Scanning electronic microscopy. Measuring interval 50 µm.

**Figure 14:**
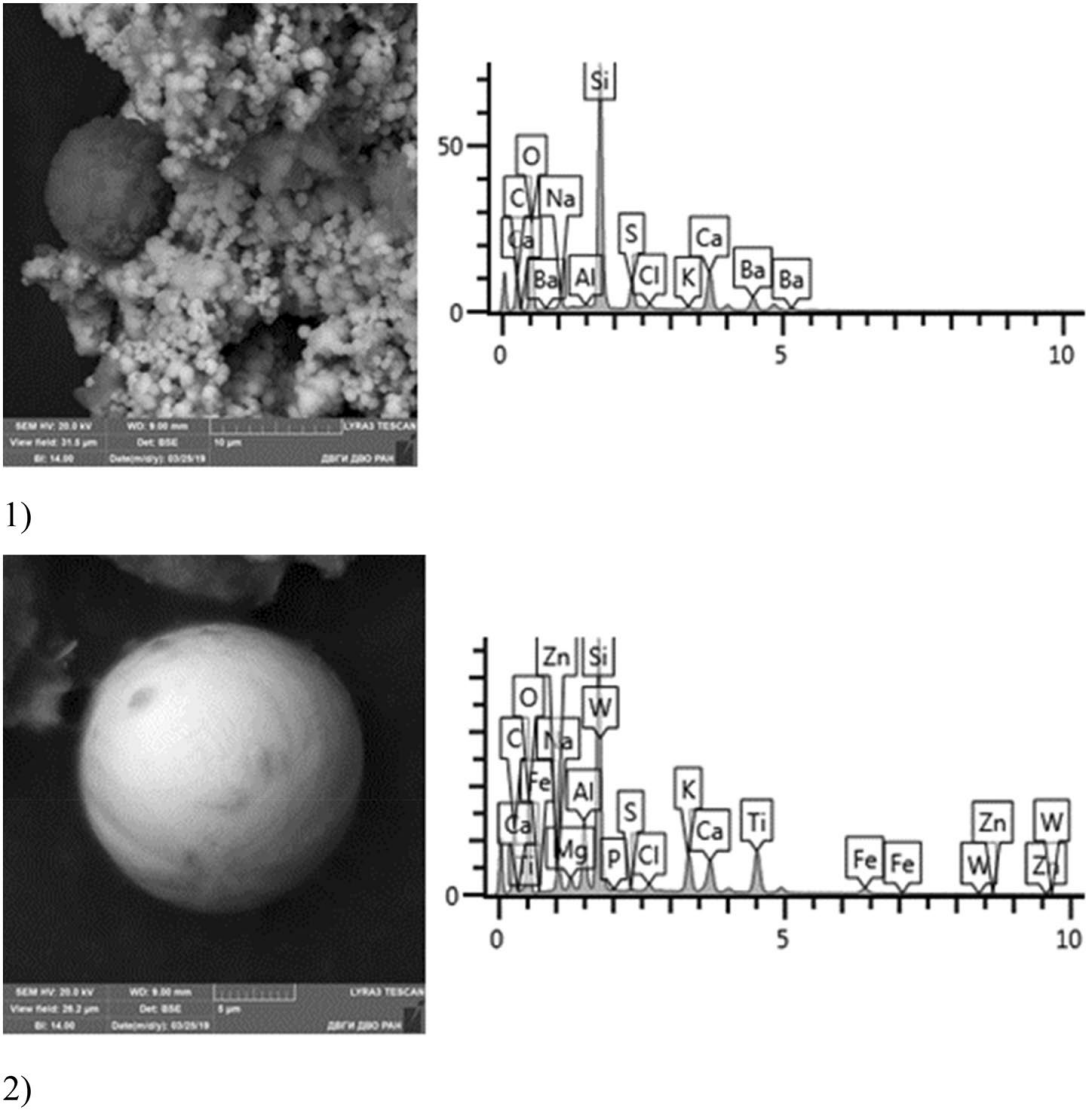
Chromium plating. Particles morphology. Scanning electronic microscopy. 1) Measuring interval 50 µm, 2) Measuring interval 5 µm.

**Figure 15:**
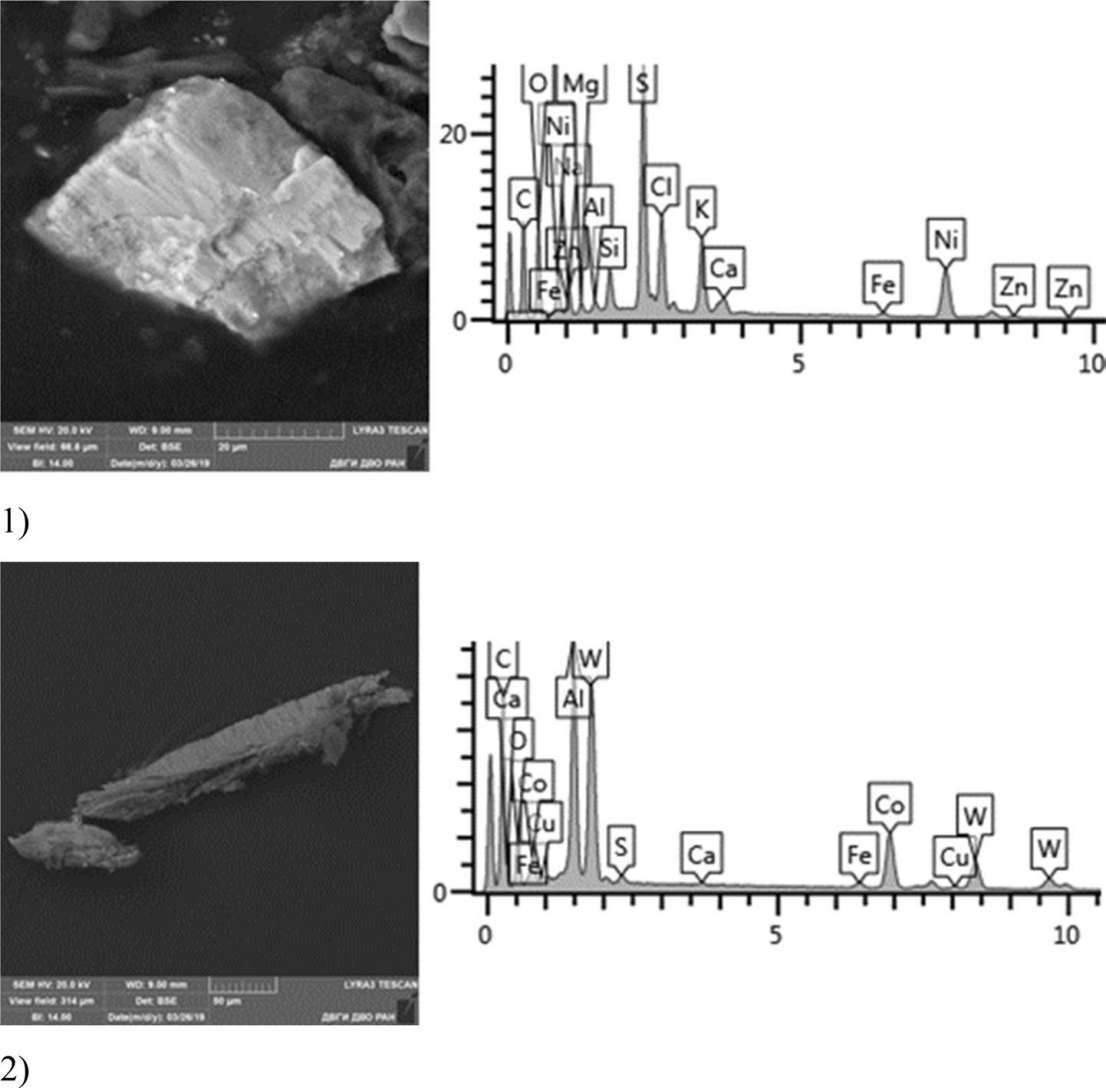
Nickel plating. Particles morphology. Scanning electronic microscopy. 1) Measuring interval 20 µm, 2) Measuring interval 50 µm.

**Figure 16:**
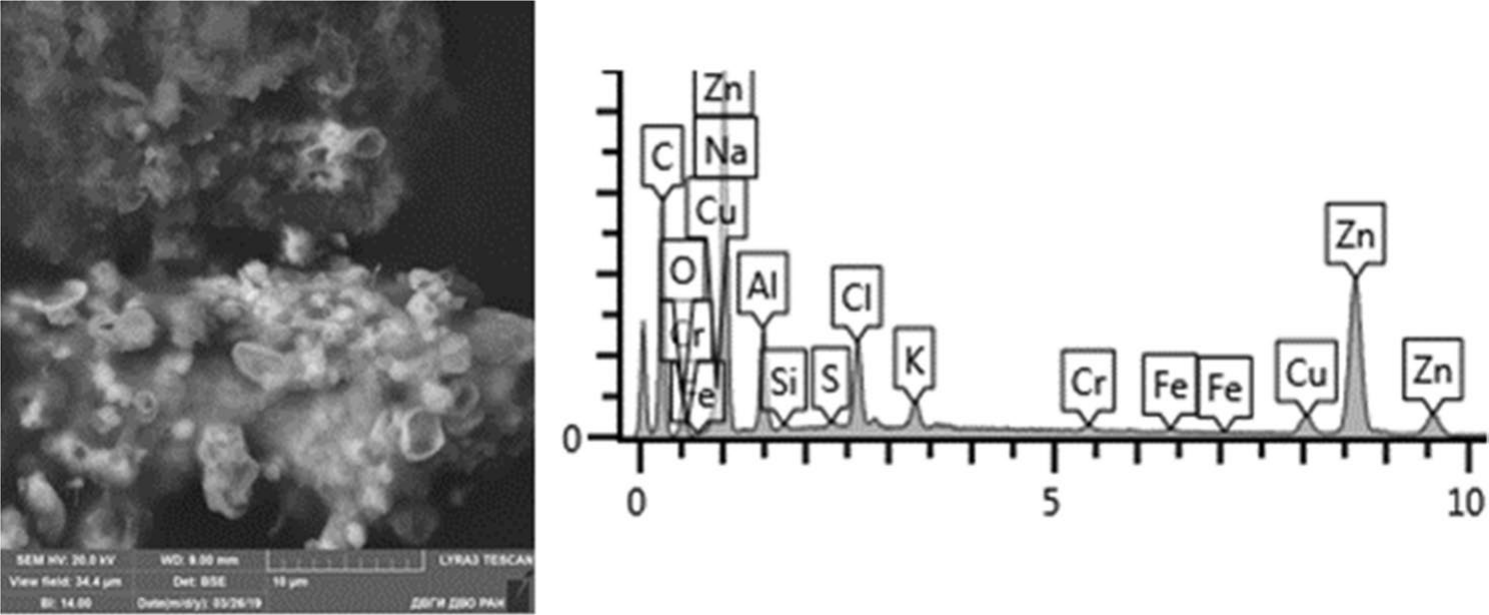
Chemical nickel plating. Particles morphology. Scanning electronic microscopy. Measuring interval 10 µm.

**Figure 17:**
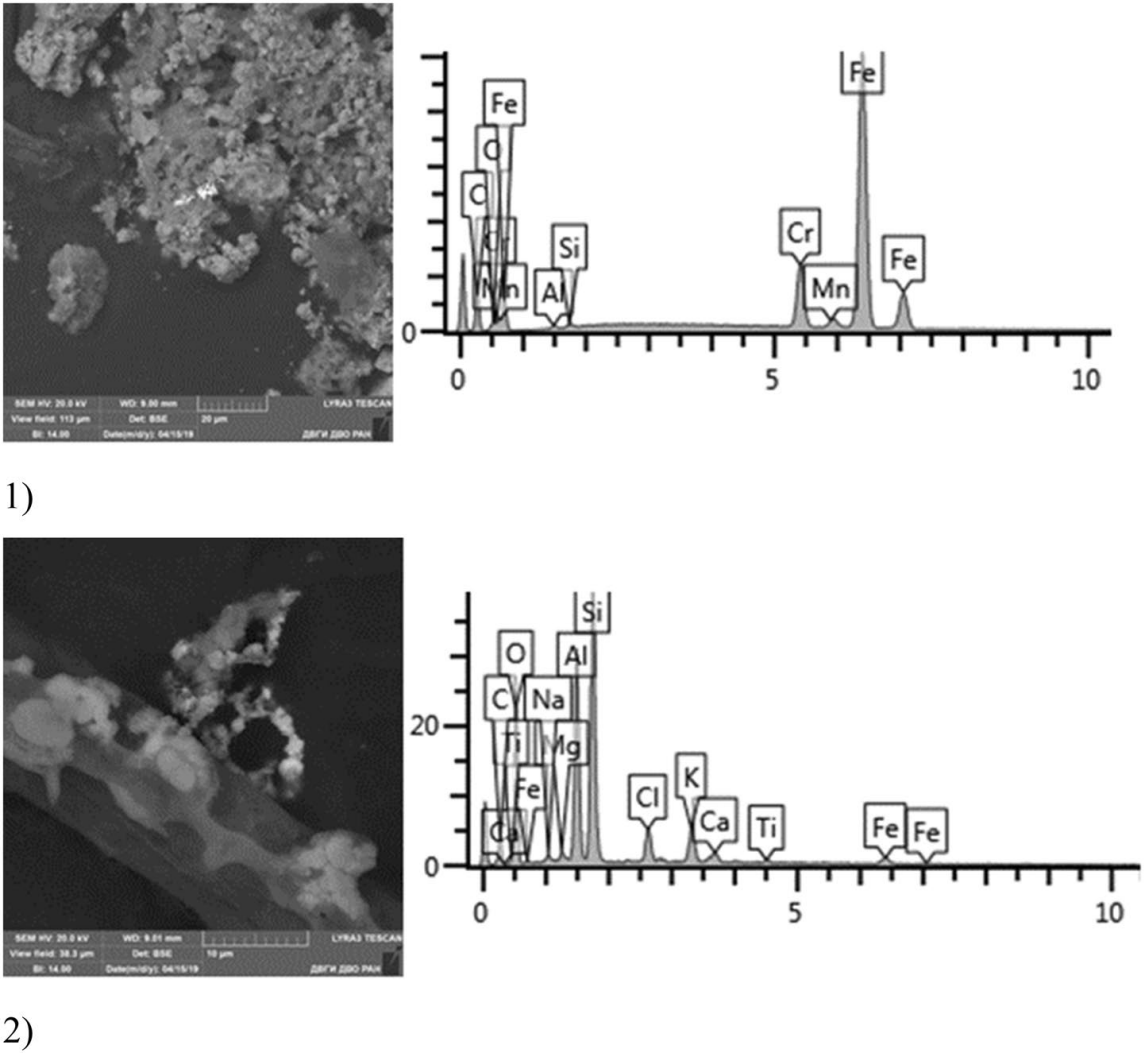
Cadmium plating. Particles morphology. Scanning electronic microscopy. 1) Measuring interval 20 µm, 2) measuring interval 10 µm.

**Figure 18:**
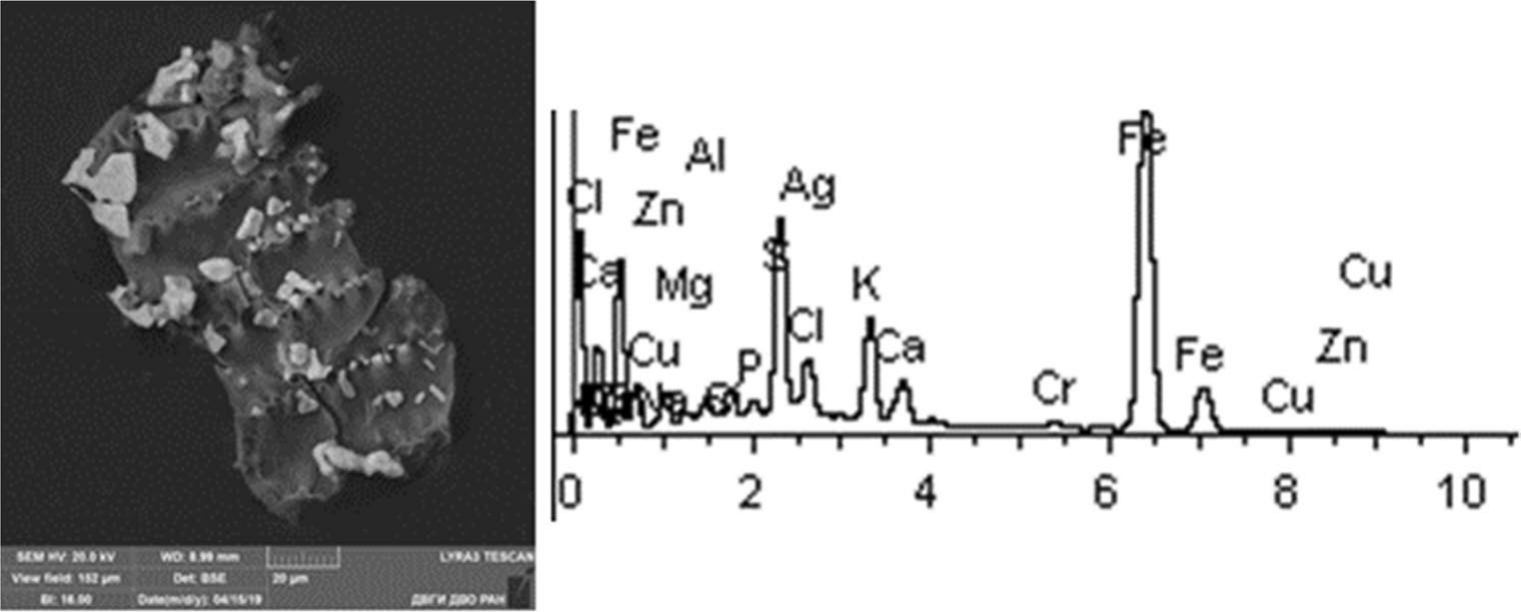
Silvering. Particles morphology. Scanning electronic microscopy. 1) Measuring interval 20 µm.

Morphologically, aluminum cleaning particles have spheroidal and a lamellar structure. Individual spheroid particles reach sizes of several tens of microns. Spectral analysis revealed high content of potassium compounds: Al, Ca, Na, Mg, Fe, Zn (chemical salts) at the level of 15–50% of the total content. The content of Zn particles (hazard class I) is 3%.

Morphological structure of aluminum etching particles in this sample is characterized by the presence of primary acicular particles forming cluster compounds. Spectral analysis revealed high content of magnesium and potassium compounds: Al, Ca, Na, Cl, Fe with inclusions of Br and S. High content of Ba particles (hazard class III) up to 30–40% is registered.

Sulfuric acid anodizing particles in this sample have varied geometric structure; there are spherical, acicular and acute-angular particles. The prevailing content of Fe particles was revealed—at 70%. These are mainly Fe contained compounds. There are compounds of S—17% and Ti—28%. It is important to note the presence of heavy metal particles of the second hazard class—Cr (from 1 to 11%), Cu (at 1–2%).

Solid aluminum degreasing particles are in the form of clusters and agglomerates, which are plate-shaped, interspersed with spherical iron oxide particles (from 10 to 30%). There are compounds of K, Fe, Na (from 10 to 20%) and Si (from 3 to 20%). The content of the elements of the first hazard class: Cd is in the range from 1 to 2%, Zn—2%. The elements of the second hazard class: Cu from 1 to 16%, Ni from 2 to 5%.

Chemical degreasing particles are in the form of agglomerates, drop-shaped clusters. The most common compounds are Na, S, (Cr, Zn, Cu), less commonly oxides of iron and aluminum, single flakes of aluminosilicates and NaCl. The predominant compounds are S, Na, and K (from 15 to 30%). The image clearly shows a large particle of Al oxide of bright white color and a particle of Fe oxide in the image below. According to the results of chemical composition analysis, the presence of heavy metals of the first hazard class (Zn) was found in the range from 1 to 4%; and the second hazard class (Cu and Cr)—from 1 to 5%.

The cathodic degreasing sample contains dendritic particles, less often lamellar with acute-angled edges. The main composition of particles is Na-S-O compounds with different contents of Cu and Zn. Singular flakes of Pb, S. The content of particles of the 1st hazard class heavy metals (extremely hazardous) Zn and Pb ranged from 3 to 11%, respectively. There are also Cu particles (hazard class II)—up to 15%.

Cold rinse particles are of grain shape, they are characterized by the presence of acute-angled facets. Most of the grains studied are iron and aluminum oxides. There are rare aluminosilicates, of iron and chromium compounds. The prevalence of Al content is characteristic—from 30 to 80% in the form of Al_2_O_3_ compounds. There are iron compounds—Fe and K.

Morphological structure is characterized by the presence of conglomerates of desmutting and metals etching particles and large dendritic aggregates. There are microinclusions of Cu, Zn, Fe, Ni, Pb and Al in various percentages, as well as inclusions of SnO and PbS. There are separate spherical particles of tin (Sn) with the content from 8 to 24%. The content of the 1st hazard class elements: Zn—from 1 to 7%, and Pb—from 2 to 48%; 2nd hazard class: Cu—8–15%, Ni—1%.

A distinctive feature of the morphological structure of chromium plating particles in this sample is the absolute dominance of spherical particles that form larger aggregates—clusters. Both iron-bearing minerals and native iron are identified. The sample contains Zr, Si, Ba, S, Fe, W, Zn, Ca, Ti. The bulk is represented by Na, Al, Si, Fe, Ti, Ca. The content of toxic elements of the 1st hazard class: Zn—1–10%, 2nd hazard class: low content of Cr particles in the sample should be emphasized—from 0.5 to 1%, Cu—1%, Co particles up to 5% and Mo—1.63%.

Morphological structure of this sample is represented by nickel plating particles in the form of polygons with relatively smooth surface. Inclusions of Ag, Fe, Cr, Ni, W, Co, Ni, S are identified. The bulk is represented by flakes of Na, S, Al, Cu, Na, Cl. Microinclusions with Ag content up to 70% and W up to 55% are registered. The content of highly toxic elements of the 1st hazard class: Zn—1%; 2nd hazard class: Ni—2–20%, Cu—up to 5%, Co—up to 18%.

Particles in this sample form larger clusters of primary particles, which confirms the data obtained from the quantitative and particle size analysis performed earlier. A Pt(Cu) microparticle was found on a carbon adhesive tape. Micro flakes of compounds of Ni(Fe), Zn, W, Fe, Cr, Pb, Cr (chemical salts), Fe, Cr, Mn, Ni, Sn were found. The matrix consists mainly of Al, Na, Cl, Pb, Cr. We also found compounds of Sn and W. The content of heavy metals of the 1st hazard class: Zn—from 1 to 40%, Pb—up to 1%; 2nd class: Cu—2–8%, Ni—from 2 to 80%, Cr—from 0.5 to 19%.

Primary cadmium plating particles in this sample form chains and numerous clusters. Spectral analysis revealed varied chemical composition; the following elements were found: SiO_2_, Al, Mg, Cl, Ca, Ti, Fe, Na, Ag, etc. The bulk of the particles deposited on the adhesive tape is represented by Fe, Cl and Al flakes; Na, Cl and Si are less common. Micro flakes of Ag, Cl, Ce, Fe, W, Cu, Fe, Cr, Ba, S were also detected.

Noteworthy is the counterintuitive fact of the absence of Cd in the sample taken near the electroplating bath with cadmium plating. The content of highly hazardous particles of 1st class heavy metals: Zn—from 1 to 8%; 2nd class: Cu—1–58%, Cr—12%, Mo—1.28%.

Morphological structure of the silvering particles has a characteristic feature, since it is a conglomerate: SiO_2_, K_2_O compounds with distinctly visible inclusions of Fe, Cr, Ag (up to 10%). Particle morphology is diverse: dendritic, drop-shaped, shaped crystals. The bulk of particles are represented by KCl compounds. Various compounds of iron Fe, Cr tungsten W, Al, S and aluminum are very common. Silver compounds Ag, Fe are a rare occurrence. Some content of Rh is detected at 2%. The content of heavy metals class of the 1st hazard class: Zn—from 1 to 3%; 2nd hazard class: Cu—1%, Ni—1–7%, Cr—0.5–15%.

The process of microscopic analysis of solid particles revealed large aggregates and clusters of primary particles of industrial aerosol. These observations confirm the growth of geometric dimensions of particles which was revealed as a result of quantitative analysis of primary particles by a laser counter and particle size analysis of settled particles collected after the end of a work shift. The evidence of particles increasing their dimensions over time due to adhesion of primary particulates and forming larger aggregates while being suspended in the air is clearly demonstrated.

Morphological analysis illustrates the invariance of the structure of technogenic particles depending on the type of electrochemical process. The presence of acute-angled particles indicates their cytotoxicity and ability to damage the human internal tissues by inhalation.

The heterogeneity of the content of chemical elements is predetermined by technological features of electrochemical processes inside the electroplating workshop and various applications of electroplating baths for details made of aluminum and other non-ferrous metals. In addition, it should be noted that heterogeneous chemical composition and changes in the size distribution of solid particles are due to physicochemical transformations of primary particles while they are suspended in the air^23^.

## Conclusions

Quantitative and mass concentrations of particulates inside an electroplating workshop were studied. Despite high quantities of particulates, the exceedance of maximum permissible concentration was detected only near the electroplating bath for cadmium plating. We can assume that the portable particle counter records gas vapor particles formed during the evaporation of the electroplating bath contents.

The absolute predominance of minute PM_0.3_ particles (data obtained using a handheld particle counter) and their further quantitative reduction (according to the measurement of particle size distribution) is associated with the aggregation of airborne particles during the work shift and the predominance of gas component of industrial aerosol.

According to the data obtained, the morphology of the surface of particles formed during various technological processes is of non-uniform structure. We observed particles of rounded shape, various agglomerates with complex geometric shapes, acute-angular particles, which when inhaled pose a maximum threat to human health.

Chemical analysis of these particles showed an absolute predominance of oxides of non-ferrous metals, the percentage of which varied depending on the type of electroplating bath. The content of highly hazardous substances of the 1st (Zn, Pb, and Cd) and the 2nd (Cu, Cr, Ni, Co, and Mo) hazard classes in each sample was recorded. The highest content of elements of heavy metals ranked in the Russian Federation as extremely dangerous for the environment (Order of the Ministry of Natural Resources No. 536 dated 04.12.2014) with a very high degree of exposure was found in electroplating baths with the following processes: cathodic degreasing, desmutting, and chemical nickel plating.

Minimizing the pollution of the working zone air with highly toxic substances generated by electroplating requires widespread use of ventilating devices and drip traps in the immediate vicinity of the electrolyte surface.

Our subsequent work will deal with detailed description of the properties of particles from each individual electrochemical process, including a series of experiments to determine the level of toxicological effects of technogenic particles on living organisms, which we will present in future manuscripts.

## Data Availability

We have no any supplementary materials.
